# Economics of Artificial Intelligence in Healthcare: Diagnosis vs. Treatment

**DOI:** 10.3390/healthcare10122493

**Published:** 2022-12-09

**Authors:** Narendra N. Khanna, Mahesh A. Maindarkar, Vijay Viswanathan, Jose Fernandes E Fernandes, Sudip Paul, Mrinalini Bhagawati, Puneet Ahluwalia, Zoltan Ruzsa, Aditya Sharma, Raghu Kolluri, Inder M. Singh, John R. Laird, Mostafa Fatemi, Azra Alizad, Luca Saba, Vikas Agarwal, Aman Sharma, Jagjit S. Teji, Mustafa Al-Maini, Vijay Rathore, Subbaram Naidu, Kiera Liblik, Amer M. Johri, Monika Turk, Lopamudra Mohanty, David W. Sobel, Martin Miner, Klaudija Viskovic, George Tsoulfas, Athanasios D. Protogerou, George D. Kitas, Mostafa M. Fouda, Seemant Chaturvedi, Mannudeep K. Kalra, Jasjit S. Suri

**Affiliations:** 1 Department of Cardiology, Indraprastha APOLLO Hospitals, New Delhi 110001, India; 2 Stroke Monitoring and Diagnostic Division, AtheroPoint™, Roseville, CA 95661, USA; 3 Department of Biomedical Engineering, North Eastern Hill University, Shillong 793022, India; 4 MV Diabetes Centre, Royapuram, Chennai 600013, India; 5 Department of Vascular Surgery, University of Lisbon, 1649-004 Lisbon, Portugal; 6 Max Institute of Cancer Care, Max Super Specialty Hospital, New Delhi 110017, India; 7 Invasive Cardiology Division, Faculty of Medicine, University of Szeged, 6720 Szeged, Hungary; 8 Division of Cardiovascular Medicine, University of Virginia, Charlottesville, VA 22904, USA; 9 Ohio Health Heart and Vascular, Columbus, OH 43214, USA; 10 Heart and Vascular Institute, Adventist Health St. Helena, St. Helena, CA 94574, USA; 11 Department of Physiology & Biomedical Engineering, Mayo Clinic College of Medicine and Science, Rochester, MN 55905, USA; 12 Department of Radiology, Mayo Clinic College of Medicine and Science, Rochester, MN 55905, USA; 13 Department of Radiology, Azienda Ospedaliero Universitaria, 40138 Cagliari, Italy; 14 Department of Immunology, SGPGIMS, Lucknow 226014, India; 15 Ann and Robert H. Lurie Children’s Hospital of Chicago, Chicago, IL 60611, USA; 16 Allergy, Clinical Immunology and Rheumatology Institute, Toronto, ON L4Z 4C4, Canada; 17 AtheroPoint LLC, Roseville, CA 95661, USA; 18 Electrical Engineering Department, University of Minnesota, Duluth, MN 55812, USA; 19 Department of Medicine, Division of Cardiology, Queen’s University, Kingston, ON K7L 3N6, Canada; 20 The Hanse-Wissenschaftskolleg Institute for Advanced Study, 27753 Delmenhorst, Germany; 21 Department of Computer Science, ABES Engineering College, Ghaziabad 201009, India; 22 Rheumatology Unit, National Kapodistrian University of Athens, 15772 Athens, Greece; 23 Men’s Health Centre, Miriam Hospital Providence, Providence, RI 02906, USA; 24 Department of Radiology and Ultrasound, University Hospital for Infectious Diseases, 10000 Zagreb, Croatia; 25 Department of Surgery, Aristoteleion University of Thessaloniki, 54124 Thessaloniki, Greece; 26 Cardiovascular Prevention and Research Unit, Department of Pathophysiology, National & Kapodistrian University of Athens, 15772 Athens, Greece; 27 Academic Affairs, Dudley Group NHS Foundation Trust, Dudley DY1 2HQ, UK; 28 Arthritis Research UK Epidemiology Unit, Manchester University, Manchester M13 9PL, UK; 29 Department of Electrical and Computer Engineering, Idaho State University, Pocatello, ID 83209, USA; 30 Department of Neurology & Stroke Program, University of Maryland School of Medicine, Baltimore, MD 21201, USA; 31 Department of Radiology, Harvard Medical School, Boston, MA 02115, USA

**Keywords:** artificial intelligence, deep learning, machine learning, diagnosis, treatment, cost-effectiveness, health economics, AI pruning, AI explainability, AI bias, recommendations

## Abstract

*Motivation*: The price of medical treatment continues to rise due to (i) an increasing population; (ii) an aging human growth; (iii) disease prevalence; (iv) a rise in the frequency of patients that utilize health care services; and (v) increase in the price. *Objective:* Artificial Intelligence (AI) is already well-known for its superiority in various healthcare applications, including the segmentation of lesions in images, speech recognition, smartphone personal assistants, navigation, ride-sharing apps, and many more. Our study is based on two hypotheses: (i) AI offers more economic solutions compared to conventional methods; (ii) AI treatment offers stronger economics compared to AI diagnosis. This novel study aims to evaluate AI technology in the context of healthcare costs, namely in the areas of diagnosis and treatment, and then compare it to the traditional or non-AI-based approaches. *Methodology:* PRISMA was used to select the best 200 studies for AI in healthcare with a primary focus on cost reduction, especially towards diagnosis and treatment. We defined the diagnosis and treatment architectures, investigated their characteristics, and categorized the roles that AI plays in the diagnostic and therapeutic paradigms. We experimented with various combinations of different assumptions by integrating AI and then comparing it against conventional costs. Lastly, we dwell on three powerful future concepts of AI, namely, pruning, bias, explainability, and regulatory approvals of AI systems. *Conclusions:* The model shows tremendous cost savings using AI tools in diagnosis and treatment. The economics of AI can be improved by incorporating pruning, reduction in AI bias, explainability, and regulatory approvals.

## 1. Introduction

The United States is the leading nation in cutting-edge medical training, research, and technology, notably in the healthcare industry. However, with the lowest health results and subpar public services when contrasted to the top ten nations, healthcare expenditure in the United States stands out as being the highest (when compared to Canada, Germany, United Kingdom, Australia, Japan, Denmark, France, the Netherlands, Switzerland, and Sweden). Between 1960 and 2022, healthcare spending in the United States increased from 5.0 to 17.9 percent of GDP (or USD 3.5 trillion), with an average increase of USD 146 to USD 10,739 per person. Almost a quarter of all healthcare dollars spent in the United States were wasted [1]. The leading causes of this expenditure include avoidable and correctable system drawbacks, such as subpar vigilance delivery, overtreatment, and improper health care delivery. This is more serious than it seems [2,3,4].

Artificial Intelligence (AI) based systems, in contrast, can dramatically reduce such inefficiencies, resulting in a considerably more efficient and cost-effective health ecosystem [5]. The incorporation of technology into healthcare has altered how we think about patient safety, hospital administration, producing new and better drugs, and, finally, making treatment decisions exclusively on data [6]. Technology has beneficial aspects for healthcare, particularly in both diagnosis and treatment [7,8]. By enabling real-time patient information to be accessed with only a few taps on a screen, technology is now paving the way for fast care management that will, in an emergency, reduce casualties. The Internet of Medical Technology (IoMT), artificial intelligence (AI), machine learning (ML), and deep learning (DL) are currently the primary drivers [8,9]. Innovation is becoming the centerpiece [10,11,12,13]. AI technological development improves existing systems, especially medical imaging [9,14] and coronary artery disease diagnosis [15,16], reducing human error, increasing patient care overall, and making doctors’ responsibilities easier [17].

The healthcare information technology (IT) sector has been driven to provide better treatments using big data, virtual reality, mobile technology, wearable medical devices, telehealth, and more, simply out of a desire to perform better [18,19,20]. The ability to reduce workflow and refocus most of a doctor’s attention on providing outstanding patient care has been made possible by systems that use AI and better data management [21]. It is impossible to overstate the value of technology in healthcare. Technical advancements has changed the face of the healthcare sector [22], and in particular, AI has changed the healthcare sector scenario. Medical applications have made extensive use of ML and DL algorithms [23,24]. AI-based solutions use databases to make decisions and are data-driven. It discovers non-linear correlations between the cardiovascular outcomes and the input predictors [25]. ML-based algorithms have the potential to simultaneously employ complicated, non-linear correlations among several input risk predictors (or qualities), in contrast to conventional statistical risk prediction methods [25,26]. For example, wall tissue characterization of atherosclerotic carotid [27,28,29,30,31,32], image segmentation [33,34,35,36,37], and cardiovascular disease (CVD) risk stratification [38] are features that DL algorithms directly extract from the input data to make predictions [9].

It has also been shown that convolution neural network (CNN) DL algorithms can extract features, followed by the training and testing of an ML-based classifier to produce a superior classification [39,40]. Recently, CVD risk and coronary artery calcium scores have been predicted using retinal images [41,42]. Predictions of diabetic retinopathy (DR) have been made using ML and DL-based systems [43,44,45,46]. Therefore, AI-based systems make it possible to examine the risk of stroke and CVD diseases and the need for human intervention [47]. The use of AI-based algorithms in specific carotid ultrasonography applications has shown promise [48,49]. Therefore, these AI-based models may be used in patient risk evaluation to jointly treat diabetic retinopathy (DR) and CVD illnesses [50].

The positive economic effect is a critical decision element in determining whether to invest in an AI solution in the healthcare business [51]. The healthcare provider and insurance businesses, in addition to the medical and pharmaceutical technology sectors, are significantly affected [52,53,54,55,56,57,58,59,60,61,62,63,64,65,66,67,68,69]. However, the broad economic impact of digital health solutions, in general, has been extensively studied in the presented paper. The saving of time in diagnosis and treatment procedures results in a direct saving of money. Using this hypothesis, an AI-based economic model for diagnosis and treatment is presented.

## 2. Background Literature

AI-enabled devices, such as advanced computed tomography (CT) scans, magnetic resonance imaging (MRIs), and ultrasounds, can carry out repetitive, simple tasks more accurately, reducing medical errors, reducing cost, and promoting early diagnosis and intervention before serious situations arise [1,2]. For instance, an Israeli start-up has created AI algorithms for diagnosing conditions including osteoporosis, brain hemorrhage, malignant tissue in breast mammography, and coronary aneurysms that are equally accurate or more accurate than humans [3]. These are powerful paradigms for preventing manual and time-consuming procedures, thereby reducing costs. According to a recent Newsweek article, AI has demonstrated 99% accuracy and is substantially faster than humans in evaluating and analyzing mammograms. This has made it possible to diagnose breast cancer more quickly, improving the cost of diagnosis [4,5].

In today’s time, the ability to precisely and successfully utilize the potential of data has authorized more effective decision making across the majority of businesses [6]. The same is true for healthcare, where massive data collection is made available for AI-enabled algorithms that can examine pattern-based outcomes, leading to improved time analysis for decision making [7,8]. Healthcare professionals are beginning to move toward AI-based solutions for predicting outcomes which can help in optimal medications based on patient profiles, thereby lowering long-term costs [9,10]. By ensuring that the appropriate actions and treatments are tailored to each patient, AI enhances clinical decision-making and provides customized care [11]. The results will be significantly improved immediately, lowering expenses related to post-treatment problems, which are a significant cost factor in most healthcare ecosystems worldwide [12].

AI has the potential to speed up the creation of life-saving medications, thereby saving billions of dollars that could be invested in maintaining healthy ecosystems [13]. A start-up supported by the University of Toronto recently created a supercomputer-based algorithm that resembles and evaluates millions of potential medications that help in forecasting their effectiveness against the Ebola virus. This directly helps in cutting costs, reducing time, and, more importantly, saving lives by reconfiguring the existing treatments [14]. Advancements in gene-based biomarkers, where billions of patient information points can be analyzed in a short amount of time from a blood sample using at-home devices, can improve AI-based drug research for clinical trials, directly affecting drug costs [15].

People can be empowered by AI to make wiser health choices. All across the world, numerous people already utilize wearable devices to collect everyday data, including heart rate and sleep habits [16]. With the help of this AI data, people at risk of particular diseases could be risk stratified well before the threat becomes acute, thereby eventually reducing cost [17]. Already, AI-based smartphone apps provide fine-grained patient profile details, which could help patients with certain chronic conditions to manage their sickness, leading to healthier lives [18]. This has a direct bearing on the economics of healthcare.

It is vital to investigate if the economic models truly meet the quality requirements that have been established to enable the decision-making for the deployment of AI in healthcare. Based on this economic analysis, our study will provide the knowledge necessary to decide in favor of or against the application of AI in hospitals, industry, and payer situations. In other words, it can be said how AI technology transforms in terms of costs, specifically the AI-based diagnosis and treatment paradigms in healthcare, and compare it against the current conventional (non-AI-based) approaches. This is exactly the aim of our study.

## 3. Search Strategy

**Figure 1:**
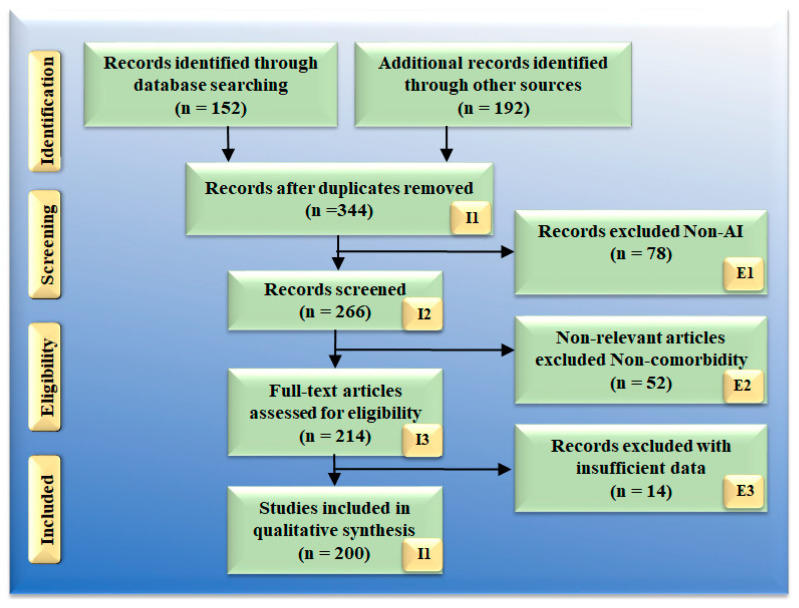
PRISMA model for selection of studies.

The Preferred Reporting Items for Systematic Reviews and Meta-Analyses (PRISMA) paradigm were used to select the 200 best AI studies for diagnosis and therapy (Figure 1) and served as the foundation for the search strategy. The usage of repositories such as PubMed, Google Scholar, and IEEE are three important databases that were utilized in the process of locating and screening relevant publications through the usage of keywords such as: “AI-based cost-effective therapy,” “AI and cost-effective treatment,” “cost-effective treatment,” “cost-effective health ecosystem,” “cost-effective AI analysis in healthcare,” “cost-effective solution in healthcare,” “AI-based cost-effective diagnosis,” “cost-effective treatment diagnosis and artificial intelligence,” “cost-effective treatment diagnosis and treatment,” “artificial intelligence,” Economics using AI,” “preventive screen using AI,” “AI-based decision making in health care,” “machine learning in health care,” and “deep learning in health care.“ Healthcare were used to exclude studies: (i) insufficient data in research, (ii) unrelated studies, and (iii) articles that were irrelevant to the topic at hand. This led to the elimination of 78, 52, and 14 studies, which were each designated by the symbols E1, E2, and E3, respectively. As a result, the final pool of research consisted of 200 different cases. Either the costs of AI-based healthcare resources are ignored in this research, or they are compared to more traditional cost models. There will be 78 studies that were not chosen to move on to the next stage of the selection process; these are denoted by the letter E1 in the PRISMA model. (i) They are not focusing most of their efforts on the economics of healthcare. For the sake of this investigation, we are only interested in works that examine the connection between AI and the economics, diagnosis, and treatment of healthcare (ii) If studies show a link between AI and cardiovascular disease, diabetes, renal disease, or any other condition, we will not consider it because there has been no cost analysis. This category, which in the PRISMA model is denoted by the letter E2 and included 52 studies, had a total of participants. The research with incomplete evidence were the ones that did not give us sufficient data to include them in our analysis. The results of these analyses indicate there is no proof to support a link between cost and healthcare resources for AI. No attempts were made to conduct such interactions. The interaction between cost analyses was not considered.

**Figure 2:**
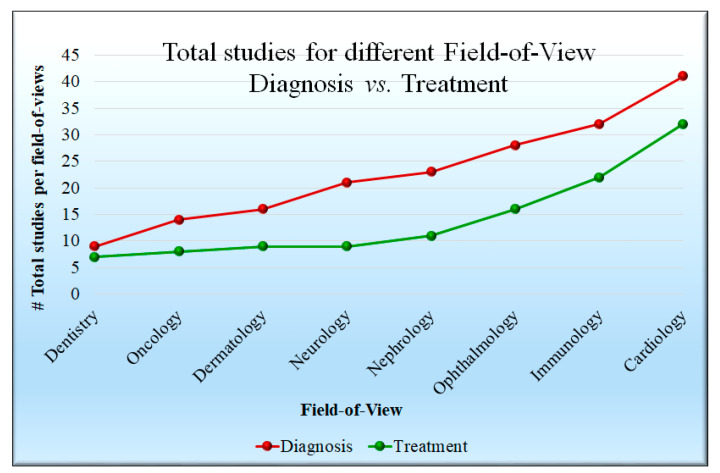
Statistical distribution of various diseases.

Studies on the diagnosis and treatment of numerous disorders, including dentistry, oncology, dermatology, kidney, ophthalmology, COVID-19, and CVD, are shown in Figure 2. Every study was subjected to a feasibility analysis before being cross-checked with scientific validation to ensure that it closely matched our objectives. Most of the papers demonstrated the role of AI in disease diagnosis with an explanation of the cost-effective technique that were available. The proposed study includes articles from various healthcare fields such as dentistry (9), oncology (14), dermatology (16), neurology (21), nephrology (23), ophthalmology focused on diabetic retinopathy (28), and immunology investigations focusing on the severity of COVID-19, which includes pulmonary. Acute Respiratory Distress Syndrome (ARDS) was shown in 32 investigations, and the studies explaining CVD, stroke severity, and risk stratification numbered 41.

The research on cost-effective AI-based diagnosis and therapy for multiple fields of view, which covers dental treatments (such as digital X-ray imaging modalities in cavity treatment, amongst other dental procedures), and several studies conclude that image-based focused radiation on lesions, targeted drug delivery, and other uses are cost-effective strategies to treat cancer [70].

The cost-effective treatment of skin illnesses such as psoriasis [71], skin cancer [72], wound care, and other similar conditions are the primary focus of a significant portion of the ongoing research. Using fog AI, it is possible to test for a wide variety of diseases at a reduced cost, including Parkinson’s [73,74,75,76], COVID-19 [77,78], and CVD [79,80]. The number of studies that demonstrate cost-effectiveness in treatment is significantly lower compared to the number of studies that demonstrate cost-effectiveness in terms of diagnosis.

## 4. An Overview of Artificial Intelligence Applications in Healthcare

The use of AI and related technologies is expanding throughout industries and sectors, including the medical field [81,82]. Providers, payers, and pharmaceutical companies all stand to benefit, which may affect a wide range of administrative and clinical processes [83]. Several studies have shown that AI is competitive with or even superior to humans at essential healthcare tasks such as illness diagnosis [31,84,85]. Algorithms are already more advanced than doctors in the diagnosis of malignant tumors and advise researchers on how to assemble cohorts for pricy clinical trials [86,87,88]. The cost factor for the AI-based system design is to be mentioned in Appendix A (Table A3). AI contains various combinations of technologies. The vast majority of these technologies have an immediate application in the field of medicine, even though the specific procedures and tasks with which they can help vary considerably [14,89,90,91,92]. The following list identifies and provides explanations for several essential AI technologies for the healthcare industry. Section 4.1 presents the AI for diagnosis systems and two classic examples of cardiovascular disease risk stratification, while Section 4.2 presents the AI-based treatment system.

### 4.1. Artificial Intelligence-Based Diagnosis Systems

**Figure 3:**
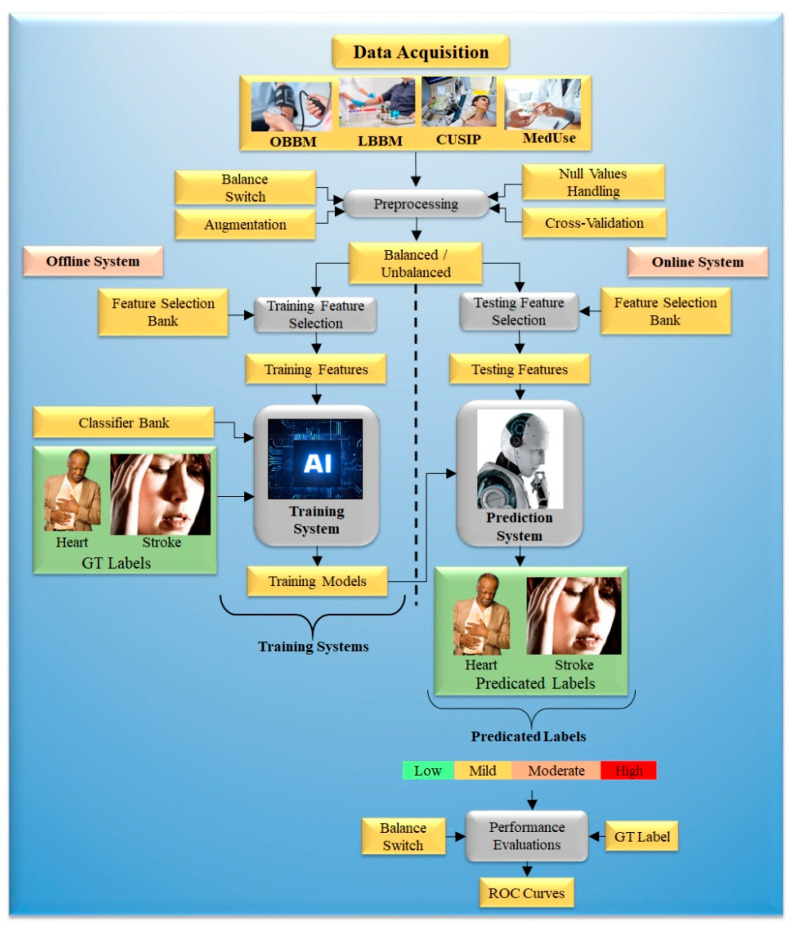
The generalized architecture of the ML-based system.

Improved risk prediction algorithms are needed to enhance overall accuracy and handle other concerns. Figure 3 depicts an ML-based system’s architecture. Generalized architecture is classified into offline and online models.

The offline model trains an ML algorithm to provide offline parameters. This will transform uncertain risk test predictors into final CVD risk labels in the online scenario [93]. The features are needed for training and label prediction in both offline and online models. Such characteristics can be derived from patient demographic trends and developed and evaluated, such as blood tests, electronic health records (EHRs), and new processes, in CVD risk analysis. Conventional CVD risk assessment calculators such as the Framingham Risk Score (FRS), Pooled Cohort Risk Equation (PCRE), and QRISK3 can accommodate a substantially greater number of possible variables than ML-based methods [94].

**Figure 4:**
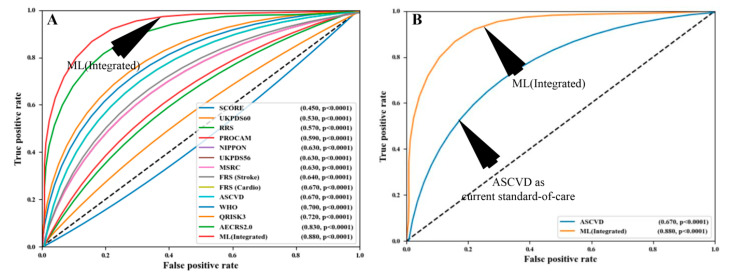
Comparing the ML-based CVD risk assessment using AtheroEdge™ 3.0_ML_ with (**A**) 13 types of CCVRC and (**B**) the standard-of-care ASCVD calculator [131].

ML-based algorithms construct the outcome, which is based on various linear and non-linear patterns found in the input risk predictors [95,96,97,98,99,100,101,102,103,104,105,106,107,108,109,110,111,112,113,114,115,116,117,118,119,120,121,122,123,124,125]. This is a critical feature of AI-driven algorithms that differentiates them from other traditional CVD risk assessments. Notable ML-based methods include support vector machines, random forests, decision trees, and extreme gradient boosting [126]. The ability to distinguish between patients with a low risk of CVD and those with a high risk of CVD is a characteristic of an ML-based algorithm [127]. In comparison to the conventional CVD risk calculators, the ML-based algorithms have provided improved risk categorization in terms of multiclass endpoints [119]. In addition to this, ML-based algorithms can identify symptomatic and asymptomatic carotid atherosclerotic plaques effectively [30,128]. Figure 4 shows the comparison of ML algorithms to statistical calculators. The AI-based algorithms were shown to have a higher total risk-strategic accuracy of 92.52% than the 13 varieties of conventional cardiovascular risk calculators (CCVRC). This was more than any of the other 13 categories combined. Others have demonstrated that ML can be used to improve risk prediction. They improved risk prediction precision by using carotid ultrasonography plaque characteristics [126,129]. Another ML-based study, conducted by Kakadiaris et al. [80] and Weng et al. [130], discovered that ML-based algorithms outperform traditional CVD risk calculators based on statistics.

**Figure 5:**
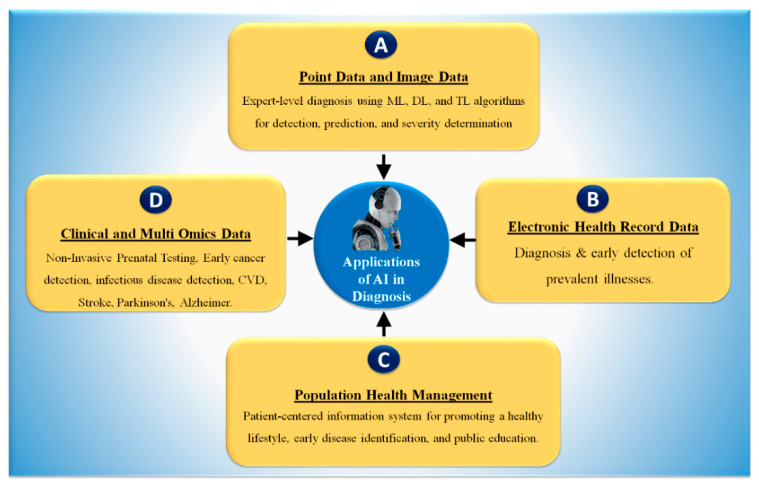
Applications of AI in healthcare diagnosis. AI: artificial intelligence, ML: machine learning, DL: deep learning, TL: transfer learning, CVD: cardiovascular diseases.

The use of AI in the diagnosis and treatment of disease has been a focus of the field at least as far back as the 1970s, when Stanford created MYCIN to detect blood-borne bacterial illnesses [93]. Model fitting and “learning” from data through model training are two critical components of ML [94]. One of the most popular forms of AI is ML, which is employed by 63% of businesses, according to a poll of 1100 US managers conducted by Deloitte in 2018 [95]. Figure 5 shows, in four sections, the basic AI technique that has led to many possible outcomes in the healthcare industry.

(A) Nowadays AI has been observed to play a significant role in computer-aided diagnosis [86,97], particularly in the identification, risk stratification, and classification of numerous diseases [8,98,99,100].

Recently, it has been explained that ML applications have dominated the field of medical imaging, including diabetes [101,102], cardiovascular disease [79,84,103], liver [98,104], thyroid [105,106], ovarian [29,107], and prostate cancers [108], as well as risk characterization using coronary and vascular screening [107,109] using carotid angiography [110]. Numerous medical imaging modalities can depict COVID-19 symptoms and lesions, in magnetic resonance imaging (MRI) [37,111], computed tomography (CT) [112], ultrasonography (US) [113], and CT for lung imaging [37,111]. (B) Using AI and key phrases, healthcare practitioners can extract patient data from faxes, clinical data, and provider notes. EHRs are lifesavers in emergencies because they give the patient’s complete medical history and allow healthcare providers to access patient data from anywhere. They improve physician and patient communication. Better communication improves care. Despite issues such as physician burnout, expenses, and lack of interoperability, EHRs can benefit the healthcare system [96]. (C) One way that AI is being used to solve the problem of medical care is to search for information in medical papers using natural language processing (NLP). This is being performed by several businesses and research groups. (D) DL algorithm can segment the COVID-19 lungs and detect the lesion in CT lung images [37,114,115,116]. As a result, we believe that AI will be effective for forecasting diagnosis and risk stratification for various diseases with good accuracy along with lower cost and shorter diagnosis time.

### 4.2. AI-Based Cardiovascular Disease Risk Stratification: A Classic Example of Diagnosis

**Figure 6:**
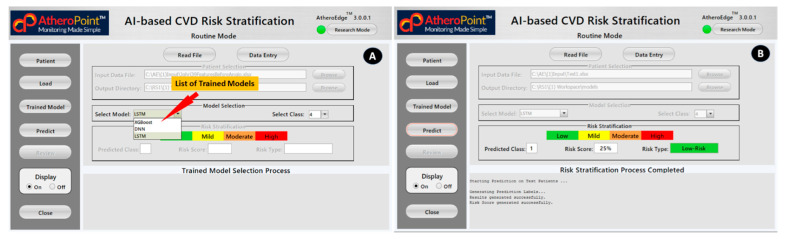
The graphical user interface of Atheropoint^TM^ (3.0) AI-based CVD Risk Stratification system to predict a person’s 10-year CVD risk. (**A**) Trained Model Selection Process and (**B**) Risk Stratification Predication Process [117]. (Courtesy of AtheroPoint, Roseville, CA, USA permission granted).

We presented an economic model that took the CVD disease into account. However, by modifying the input covariate of the model, such as Parkinson’s, diabetes, COVID-19, renal, etc., we can adjust the paradigm of the model. Economic analysis is necessary to assess resource consumption and guarantee optimal use because CVD diagnosis and treatment are costly. The American College of Cardiology (ACC) and the American Heart Association (AHA) has issued several guidelines throughout the years, the most current of which has encourage the use of specific algorithms to conduct a CVD risk assessment (Figure 6). Statin medication is typically prescribed to patients to reduce their overall risk of CVD based on the projected risk, which is calculated using risk calculators.

These shortcomings necessitate the development of a more robust and accurate model for predicting the risk of developing CVD. Incorporating image-based phenotypes into CVD risk prediction models can enhance conventional risk calculators. Suri et al. [15,118,119,120,121] have made an effort in this direction by merging traditional risk indicators with image-based phenotypes based on automated carotid ultrasonography [122]. This fusion was used for determining the 10-year CVD risk [118,123,124]. Each slice of the pie represents one of the conventional risk variables or carotid imaging phenotypes that contributes independently to the 10-year CVD risk [125,126,127,128,129,130,131]. 

### 4.3. Deep Learning-Based Diagnosis and Risk Stratification

**Figure 7:**
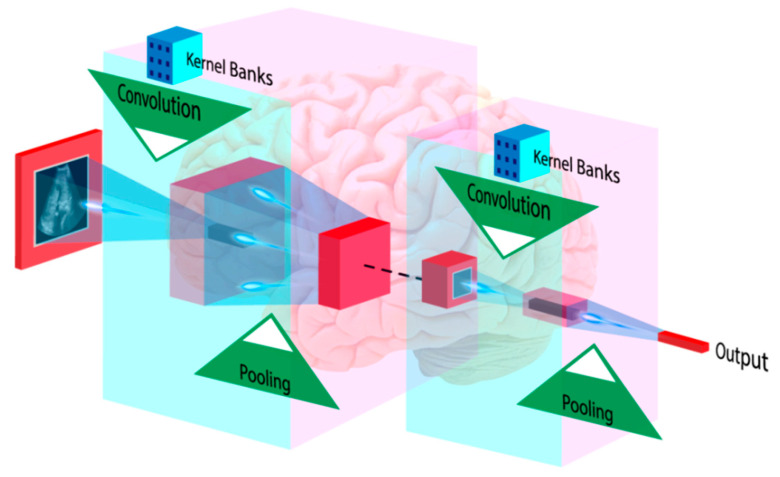
CNN-based medical image analysis architecture. (Courtesy of AtheroPoint, Roseville, CA, USA permission granted).

DL-based algorithms are also capable of making a comprehensive diagnosis. Medical image analysis can benefit from DL techniques such as classification and feature extraction [132]. DL algorithms extract their features and conduct classification or prediction [133]. In medical imaging, CNN is widespread, and DL empowers this algorithm. CNN can employ high-level features to diagnose medical conditions [39,134]. Figure 7 shows how an input image is convolved using kernels to extract high-level patterns. The pooling process chooses relevant, dominant features. During CNN training, backpropagation learns all kernel coefficients. CNN was used to classify carotid ultrasound images into lipid, fibrous, and calcified plaque [135]. CNNs are also used to measure carotid intima-media thickness and lumen diameter [136,137,138]. Rim et al. [41] employed DL to predict CAC from retinal pictures. The authors showed that retinal CAC values are equivalent to CT-derived ratings. Cheung et al. [42] assessed CVD risk using retinal vessel caliber. DL-based algorithms are also used to screen DR patients [45,46,139].

AI-based algorithms can be used for accurate CVD and DR risk assessment with established risk variables, such as carotid ultrasonography plaque phenotypes.

### 4.4. Artificial Intelligence-Based Treatment Systems

AI is needed for integrative approaches for handling complicated diseases such as cancer. Data integration includes concatenating omics data characteristics. Since biomarkers link with biological pathways, AI researchers have identified cancer subtypes and possible therapeutic radiogenomics. AI predicts disease prognosis and therapeutic responsiveness. These therapeutically relevant achievements must be more robust for customization or personalized medicine.

Radiogenomics has the potential to be leveraged as a useful technique in oncology to select the most appropriate patients [140]. This possesses the possibility of functioning as a digital, non-invasive biopsy tool that can detect and measure tumor lesions help create customized immunotherapy regimens, and enable ongoing treatment response monitoring [140]. There is reason to believe that combining imaging data with radiomics may result in improvements to disease diagnosis, prognosis, and the ability to forecast the outcomes of disease. The use of radiogenomics research in various diseases, including glioblastoma, hepatocellular carcinoma, non-small cell lung cancer, hematological tumors, and others, may provide an excellent representation of the advances made in this field [140,141].

**Figure 8:**
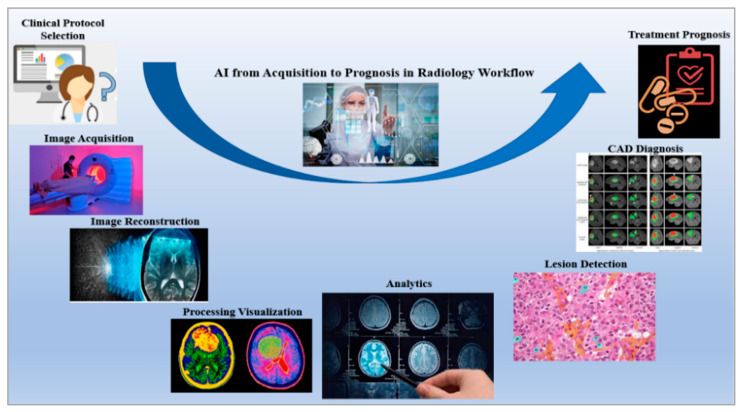
Role of AI in improving the pipeline of radiology, from clinical protocol selection to treatment prognosis [12].

The advent of radiogenomics has prompted a shift in the focus of research from the level of radiology and pathology to the level of genetics [142]. Mining radiomics, genetic data, and clinical records have contributed to the consistent expansion of the field of radiogenomics during the past ten years [141]. Research in the field of radiogenomics has significantly benefited from the development of deep learning and big data programming, which in turn has contributed to the creation of newer algorithms, workflows, and approaches [143]. The development of a completely automated system paired with a radiological workflow, such as the one represented in Figure 8 [144], is a notable breakthrough in the field of radiogenomics. This results in a reduction in the total amount of time spent performing tasks that are repetitive and laborious, while simultaneously boosting both efficiency and productivity [145,146]. Another advantage is that the treatment can be monitored in real-time by comparing many photos from the database at the same time [144,146].

The ability of radiogenomics to aid in the creation of individualized treatment plans relies heavily on the reliability and openness of its predictive tools and computer algorithms [147]. A multivariable prediction model for individual prognosis (TRIPOD) and other such recommendations have been essential in getting us closer to our aims [142]. It is crucial, however, to ensure that the implementation of such cutting-edge radiogenomics methodologies takes into account the weakness of the presently available radiobiology expertise [147]. New conclusions can be established by combining the imperfect and erroneous datasets available in the radiogenomics database with preexisting knowledge of the results [148].

## 5. Economics of Artificial Intelligence Models

**Figure 9:**
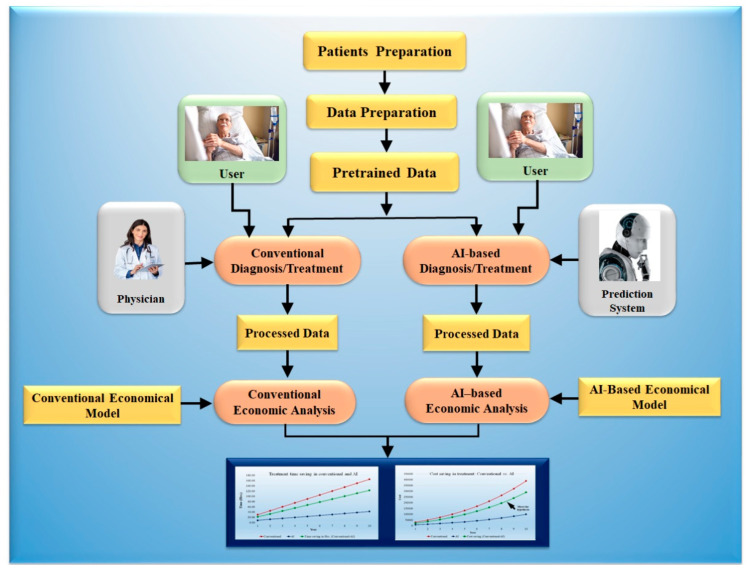
The AI economical model for the diagnosis and treatment against the conventional model.

AI reduces healthcare costs as compared to conventional methods. It has been shown before the cost saving due to AI in treatment is more effective as compared to diagnosis [149]. AI reduces time in diagnosis and treatment as compared to conventional methods. In a short time, high accuracy in diagnosis and treatment can be achieved. AI helps improve diagnostic accuracy by eliminating prejudice and subjectivity [150]. AI-based medical diagnosis reduces the likelihood of inaccurate examination. Patients may feel more at ease when seeing a doctor because of AI technology. AI filters through a considerable amount of data to determine which therapies will produce the best results. Not only can implementing AI technology in health care reduce costs, but it can also help organizations to maximize their ROI. Figure 9 shows an AI-based/conventional diagnosis and treatment model.

The AI economical model for the diagnosis and treatment has been presented. The model predicted the cost savings for 10 years. Initially, a cohort of 20 hospitals and 20 patients per hospital for a whole year were selected for the analysis. The detailed analysis is shown in Appendix A. For designing the model, we have taken an assumption for growth of 10%. The time required for diagnosis is to be assumed as per standard and compared with the AI-assisted tools. As less time is necessary for AI-based diagnosis, it saves costs.

### 5.1. Modeling Cost Analysis for Diagnosis

**Figure 10:**
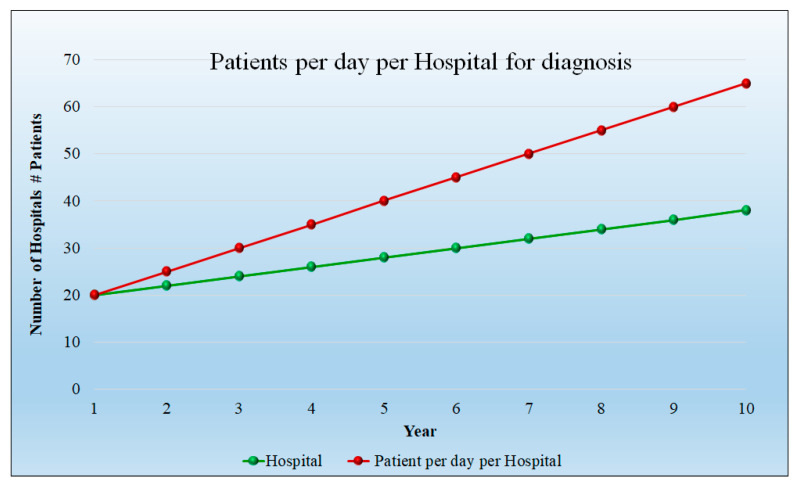
Patients per day per hospital for diagnosis.

Assumptions: For the analysis, the 10 years were considered, while for the starting year, the number of patients were 20 per day per hospital and the number of hospitals considered was also 20. The progression rises to 65 patients per day and the hospital count is 38 at 10 years. Figure 10 depicts the number of patients per day in each hospital as well as the number of hospitals. It indicates that the patient count and hospitals are increasing linearly. The detailed statistical analysis of the model is shown in Appendix A (Table A1).

**Figure 11:**
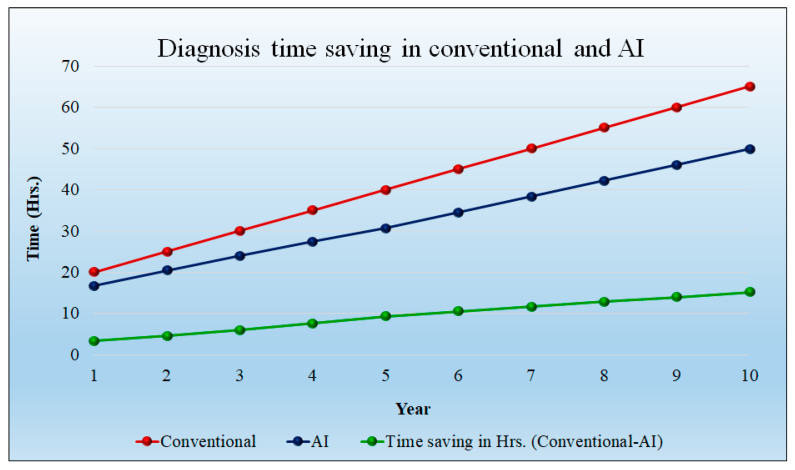
Time-saving for AI-based diagnosis model (green). Conventional model (red) vs. AI (blue) showing year vs. time (in hours).

Figure 11 shows the time saved (hours) during the diagnosis of the patient. In the initial year, the time savings is 3.33 h per day; at 10 years, the time saving will be 15.17 h per day. Over the course of a year, the savings in time increased even with the increase in patient quantity. The cost of diagnosis is reduced as a result of the time savings. The detailed statistical analysis of the model is shown in Appendix A (Table A1).

**Figure 12:**
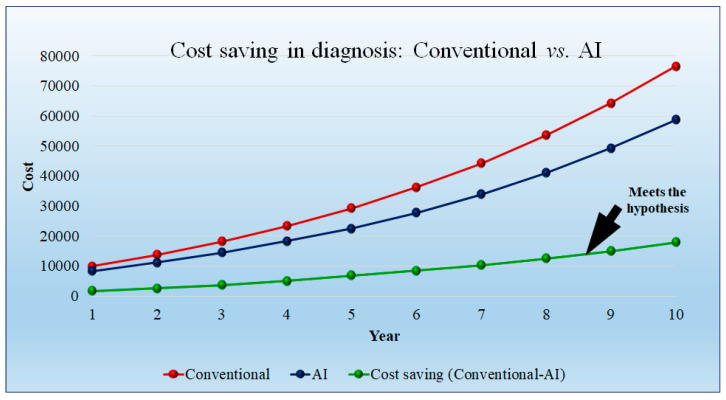
Cost saving (green) in diagnosis: conventional (red) vs. AI (blue).

Figure 12 shows the cost saving of the AI-based diagnosis method as compared to the conventional diagnosis method. The conventional diagnosis method requires greater time compared to the AI model. We assumed the model diagnosis price as USD 500 per hour. This observation results in cost saving in the initial year, but after 5 years the cost is saving is more. The cost savings in diagnosis are USD 1666.66 per day per hospital in the first year and USD 17,881 per hospital in the tenth year. The detailed statistical analysis of the model is shown in Appendix A (Table A1).

### 5.2. Modeling Cost Analysis for Treatment

**Figure 13:**
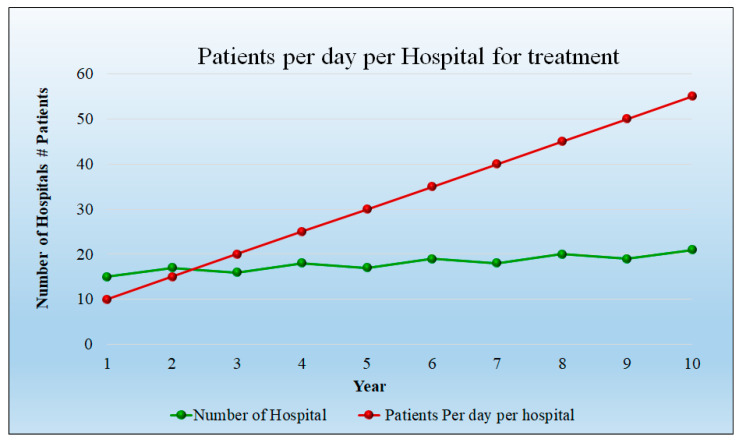
Patients per day per Hospital for treatment (red), number of hospitals (green).

The cost associated with the treatment is higher as it requires more time for prognosis. The curve in Figure 13 shows the number of patients admitted initially to hospitals and the number of hospitals. The detailed statistical analysis of the model is shown in Appendix A (Table A2).

Treatment requires more time as compared to the diagnosis. It indicates that the patient count and hospital count are increasing linearly. For the analysis purpose, over a 10-year span, we have considered an initial year of 20 patients per day per hospital; for the initial year, 15 hospitals were considered. The progression rises to 55 patients per day and the hospital count is 21 at 10 years.

**Figure 14:**
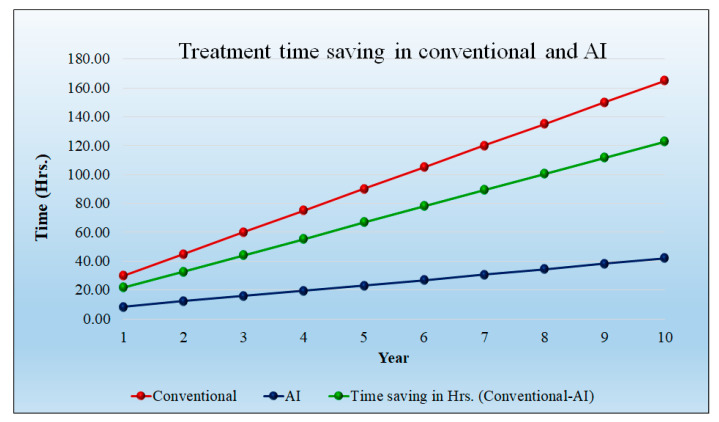
Treatment time-saving (green). Conventional time (red) and AI time (blue).

Figure 14 shows the time saving (hours) during the treatment of the patient. Note that saving in time increases even if there is an increase in the patient sample size. The time-saving results decrease in cost. The time-saving treatment in the 1st year is 21.67 h per day per hospital, and it reaches its peak in the 10th year at 122.83 h per day per hospital. The detailed statistical analysis of the model is shown in Appendix A (Table A2).

**Figure 15:**
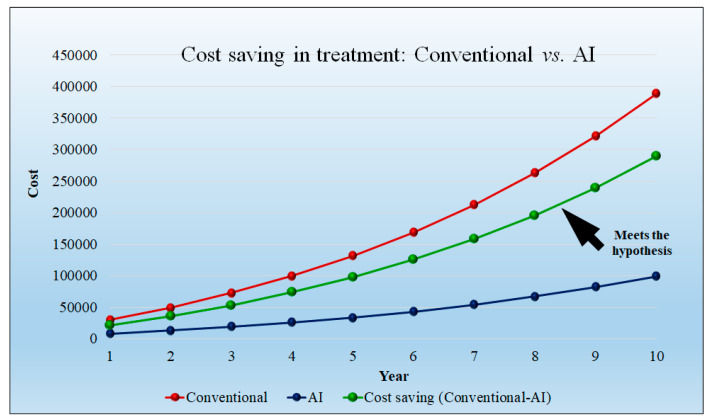
Cost saving in treatment (green) shows a non-linear curve. Conventional treatment cost (red) vs. AI treatment cost (blue).

The Figure 15 curve shows that the cost saving of the conventional treatment method requires more time, hence, the cost is higher. However, treatment using AI requires less time even with the increase in the patient quantity. We had assumed the model treatment price is USD 1000 per hour. This observation results in less cost savings in the initial year, but after 5 years, the cost saving is increased. The cost savings in treatment are USD 21,666.67 per day per hospital in the first year and USD 289,634.83 per day per hospital in the tenth year. The detailed statistical analysis of the model is shown in Appendix A (Table A2).

### 5.3. Cost Saving in USD Using AI-Based Diagnosis and Treatment Tools

**Figure 16:**
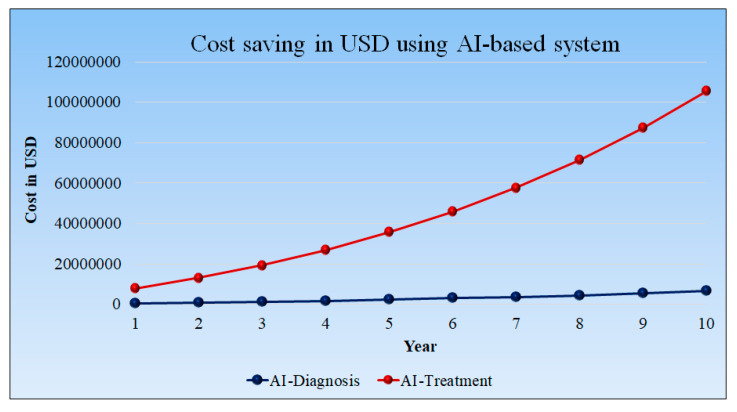
Cost saving in USD using AI-based system, AI-Diagnosis (blue), AI-Treatment (red).

Figure 16 shows the cost saving in USD using AI-based tools. However, diagnosis using AI requires less time even as the patient quantities increase. This observation results in less cost savings in the initial year, but after 10 years, the cost savings are higher.

The cost associated with the treatment is higher as it requires more time for prognosis. The time-saving treatment in the 1st year is 21.67 h per day per hospital, and it reaches its peak in the 10th year at 122.83 h per day per hospital. The cost savings in treatment are USD 21,666.67 per day per hospital in the first year and USD 289,634.83 per day per hospital in the tenth year. The detailed statistical analysis of the model is shown in Appendix A.

## 6. Recent Advances in Artificial Intelligence and Its Relationship to Economics

Three major advancements in the field of AI that cannot be neglected are, namely, (a) pruning of AI (PAI) models, (b) explainability of AI (XAI) models, and (c) Risk of Bias (RoB) in AI models. These are vital for improving AI performance and comfort level of integrating the AI models in AI-based products in diagnosis and treatments.

### 6.1. Pruned Artificial Intelligence Systems and Its Effect on Economics

ML and DL techniques have been widely used for various disease detection and classification due to their powerful ability to build training models which can be used for prediction on unseen or seen data sets [36,126,151,152,153,154,155]. Utilizing GPUs or supercomputers is one method for resolving the processing challenge [156,157,158]. Even though they are costly and challenging to maintain over time, LeCun et al. [159] were the first to introduce the idea of pruning to the field of deep learning in their 1989 publication, “Optimal Brain Damage.” Pruning is the process of eliminating extra weights from a model or query region to eliminate unnecessary or unimportant areas [160]. By selecting the appropriate and correct hyperparameters during model training, this pruning approach was expanded to optimize storage [161] and speed up model development [29].

**Figure 17:**
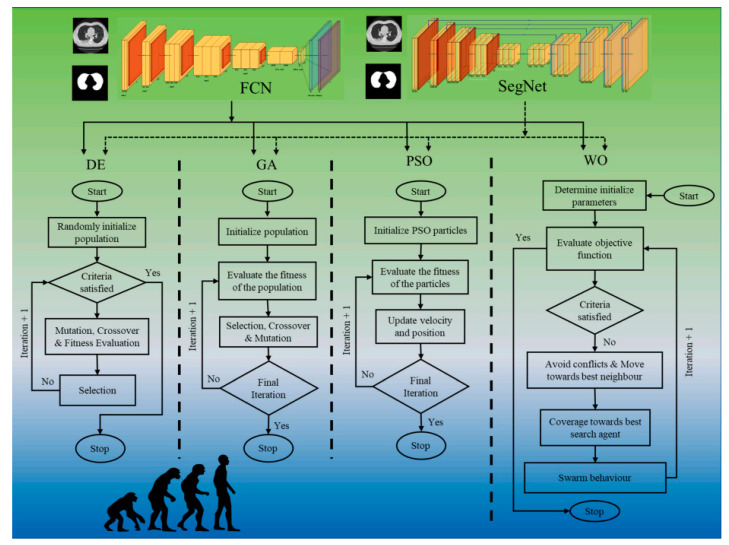
Eight systems were created using four pruning approaches (DE, GA, PSO, and WO): FCN-DE, FCN-GA, FCN-PSO, FCN-WO and SegNet-DE, SegNet-GA, SegNet-PSO, and SegNet-WO [35].

Agarwal et al. [35] implemented eight pruning deep learning models for COVID-19 computed CT lung segmentation and heat map localization images. Four evolution algorithm (EA) approaches, namely Differential Evolution (DE), Genetic Algorithm (GA), Particle Swarm Optimization (PSO), and Whale Optimization (WO) were used to optimize two basic DL networks, fully connected network (FCN)/segmentation network (SegNet), to solve the storage and speed issue (Figure 17). The eight pruning procedures are thus four times two (i) FCN-DE, (ii) FCN-GA, (iii) FCN-PSO, and (iv) FCN-WO, with FCN and (v) SegNet-DE, (vi) SegNet-GA, (vii) SegNet-PSO, and (viii) SegNet-WO in DL framework. These pruning methods need more evaluation in terms of the tradeoff between parameter size vs. real-time usage vs. performance of AI pruning models. If the performance of the pruned AI model is superior to conventional AI models, this will further improve the economics in diagnosis and treatment paradigms.

### 6.2. Explainable Artificial Intelligence Systems and Its Effect on Economics

DL techniques have drawn a lot of attention, since they frequently outperform humans in tasks such as recommendation systems, speech and image recognition, and many others. However, these applications are not reliable or comprehensible. A common misconception about DL models is that they are opaque, challenging-to-understand black boxes with complex underlying mechanisms. However, depending on the application, mistakes made by AI systems could be catastrophic. In the medical industry, the lives of the patients depend on these decisions, whereas an unmanned vehicle’s vision-based system error could result in a crash.

**Figure 18:**
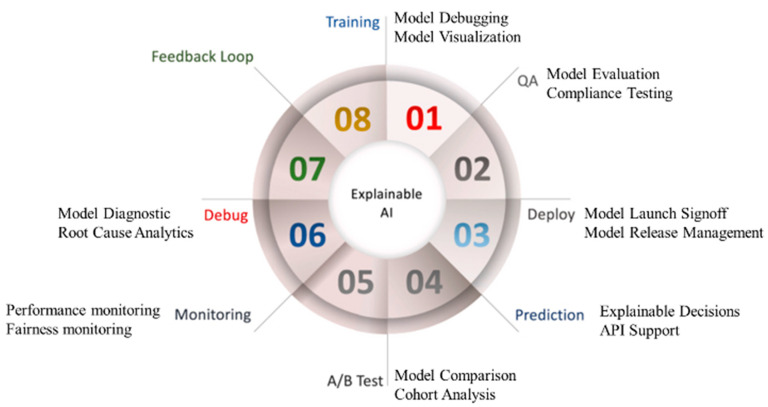
Eight aspects of Explainable AI [165].

Explainable AI (XAI), is used to solve the aforementioned challenges. Recently, scientific validation was also evaluated with the help of XAI [37,98,162,163,164]. The role of justice, privacy, openness, and explainability in the DL paradigm has been further developed by the European General Data Protection Regulation (GDPR) [165]. Figure 18 shows the seven customizable processes of DL, which are DL training, quality assurance (QA), installation/deployment, prediction, cross-validation-based testing (A/B test), monitoring, and debugging. This is possible because XAI incorporates a feedback loop. The usability of the AI system improves if XAI is incorporated into the AI system. The demand for such an XAI system grows automatically, hence more considerable revenue. Further, it provides more stability to the AI system, giving longer life to the product design.

### 6.3. Bias in Artificial Intelligence Systems and Its Economics

AI systems were recommended as a potential substitute for existing diagnosis and treatment approaches [166,167]. AI systems, on the other hand, confront several challenges, one of which is a tendency to prioritize accuracy over scientific validation and clinical evaluation [168,169]. Due to a lack of robust ground truth selection such as CVE, coronary CT score, or angiography stenosis, the disease severity ratio is typically approximated and not accurate. It places a significant emphasis on the AI system’s resilience while only a slight emphasis is placed on its authenticity [170]. It introduces bias into the AI system [13,16,155,168,169,171]. It is also important to note that the database contains specific regional patient characteristics; as a result, the model may under or overestimate diagnosis and treatment findings for different ethnicities or comorbidities [164].

Therefore, identifying risk-of-bias in artificial intelligence systems (RoB) [166,167] and adjusting the diagnosis and treatment are essential steps in the process of enhancing risk stratification in emergency department patients. By combining elements such as mobile, cloud, and e-health infrastructure, the performance of AI-based risk classification and therapy can be considerably improved.

## 7. Regulations and Artificial Intelligence-Based Systems

### 7.1. Motivation for Building AI-Based Products for a Successful Regulatory Market Approval

The challenge in today’s world of biomedical engineering system design is that the focus is on the accuracy and performance of the system, but not on the reliability, stability, safety, failure mode, robustness, sensitivity analysis, mitigation during the failure mode, ability to perform risk analysis and risk mitigation, building the contradictions, and solid user manuals. This causes the system to become unreliable and eventually die out over a little course of time. To sustain AI products, one needs time-to-time regulatory approvals and memo-to-file (MTF). Most of the diagnosis products fall in the Class II category by the FDA 510 (K) regulations. They do not require clinical trials, while most therapeutic products (especially invasive) fall in the Class III category and require clinical trials. Thus, understanding AI-based products and their link to food and drug regulation (FDA) are of vital importance.

### 7.2. What Should an AI-Based Product Undergo for a Successful FDA 510 (K) Approval?

During the AI-based system design, the following points are to be kept in mind for regulatory 510 (K) approval. This is not limited to these, but can be summarized as follows: (i) customer requirements should be clearly laid out; (ii) engineering specifications should be planned well; (iii) used cases must be established; (iv) engineering design should be carefully designed ensuring proper use of 3rd party systems, such as gold standard if the system is a supervised AI-based system; (v) solid verification and validations systems designs; (vi) user-manuals should show under what conditions the system works, the noise conditions, under what bounds the system will malfunction, and what are the mitigations under failure conditions, what are the alternatives for the clinicians (users); (vi) failure mode effective analysis (FMEA) must be duly performed along with risk analysis and risk mitigations; (vii) traceability analysis which links the customer requirements, engineering specification, engineering design, and mitigations; (viii) thorough design of contraindication and predicate designs for the 510 (K) approvals [172].

In AI-based design, AI explainability is of vital importance since most AI-based systems are black boxes; therefore one must show the explanations and justification of the results, such as where the lesions are by color codes (say heatmaps) or show which AI-based features are crucial and why, such as usage of LIME or SHAP by showing, graphically, the positive or negative side of the feature strength [173,174].

The parent firm should submit medical hardware or software for FDA review before it may be sold legally in the US market [175,176]. The regulatory body has three levels of clearance for medically focused AI/ML-based algorithms, including 510 (k), premarket approval, and the de novo pathway, each of which comes with unique requirements that must be met (Table 1).

### 7.3. A Short Note on the Influence of the Changing Technology and Economics

Technologies are constantly evolving and volatile; in the case of AI-based systems, the vulnerability is even higher. It is hard to see engineering knowledge stay in one place due to its demand, and thus the reliability of the human capital is at stake. This affects the engineering design and its reliability. The company management should incentivize the engineering resources to stabilize by ensuring a win-win situation for the long-term objectives of the industries, leading to successful regulatory 510 (K) approval and regular MTF. Thus, the factors such as changes in technology, retaining human capital, long-term goals of the companies, and FDA regulation, all are tandemly connected and cannot be ignored for a successful business model.

The FDA’s approach to dealing with repetitive revisions primarily relies on manufacturers to uphold GMLP, which stipulates that data from training and testing must be kept separate, algorithms must be evaluated for relevance, and execution and reporting by the manufacturer must be genuine and straightforward. Thus, as long as it accepts continuous advancements, this platform will provide patients with timely access to the most recent technology. However, it is crucial to consider carefully the details that manufacturers provide regarding an algorithm’s design, the intended use for which it is intended, and the effects of changes on local performance [178]. When attempting to evaluate algorithm performance reliability and consistency, there are numerous obstacles [179]. Since each company must purchase its training and testing images, the lack of uniform test sets hinders development and makes it more challenging to evaluate the data modality [180]. This can be a considerable time and expense drain, which might result in an accidental bias in the test sets favoring particular equipment manufacturers, patient groups, or the methods used by technicians to gather the scans. Itis also crucial to realize that the maker bears the responsibility for verification and integrity, which could lead to dishonest use of the technique for financial gain [181].

## 8. Discussion

### 8.1. Principal Findings

This is the first study in the field of AI economics, as well as an investigation into the cost analysis of AI models for diagnosis and treatment. The review explains how to save costs and time by adopting AI-based solutions in diagnosis and treatment. In a progressively sequential task, we analyze the cost parameter and time for diagnosis and treatment. Further, our study explains the motivation for building AI-based products for successful regulatory market approval, and further to undergo successful FDA 510 (K) approval.

We demonstrated that AI lowers healthcare costs when compared to traditional methods. The cost savings from AI in treatment are more effective than the cost savings from AI in diagnosis. When compared to traditional methods, AI saves time in diagnosis and therapy. High accuracy in diagnosis and treatment can be accomplished in a short period. AI-assisted diagnosis improves diagnostic accuracy by removing bias and subjectivity. AI-based medical diagnosis decreases the possibility of incorrect examination. Because of AI technology, patients may feel more at ease when visiting a doctor.

AI examines enormous volumes of data to determine which treatments will produce the best outcomes. Implementing AI technology in the healthcare sector can help firms maximize their returns on investments while also reducing costs. The biggest challenge facing AI in many healthcare disciplines is not whether the technologies will be advanced enough to be useful, but rather ensuring their acceptance in routine clinical practice. For AI systems to be widely adopted, they must be certified by regulatory bodies, connected with EHR systems, standardized to the point that similar products perform similarly, taught to physicians, paid for by public or commercial payer groups, and maintained over time.

### 8.2. Benchmarking

An analysis of the information shows that a few studies using different imaging modalities such as MRI, CT, X-ray, US, and ECG have been linked with AI models for the various disease diagnosis and treatment of renal, pulmonary, carotid artery disease, coronary artery disease, DR, and COVID-19. There is very little discussion of AI’s economic modeling seen in the literature. Only a few studies highlight AI models’ economic consequences and operating costs. The benchmarking Table 2 is shown for a few specific studies.

Smetherman et al. [182] explained in detail AI products in radiology, and numerous novel uses for these technologies in breast imaging. In addition to outlining potential future payment channels, the article describes the current situation of reimbursement for breast radiography AI algorithms under the conventional fee-for-service model employed by Medicare and private insurers. Additionally, the reader is given a full explanation of the inherent difficulties associated with using the current American payment system for AI radiology systems. To effectively integrate these cutting-edge technologies into their practices and increase patient care and workflow efficiency, breast radiologists are looking for a better grasp of how AI will be compensated.

Challen et al. [183] focused on the development of AI in health through the use of ML as a promising area of research, but it is challenging to determine how accurate these systems might be in clinical practice or how reproducible they are in various clinical contexts due to the rapid pace of change, diversity of different techniques, and multiplicity of tuning parameters. This is made worse by the lack of agreement over the best way to disclose a potential bias in ML studies. For this, the authors think that the Standards for Reporting of Diagnostic Accuracy effort could be a good place to start. Additionally, researchers must think about how ML models, such as scientific data sets, can be licensed and distributed to enable the replication of research findings in other contexts.

Yuan et al. [184] proposed that the evolution of medical practice from empirical medicine to evidence-based medicine, intelligent diagnosis, and AI-directed medicine is something we are currently witnessing. Although AI in medicine is still in its infancy, there is no doubt that by utilizing the diversity and complexity of real-world data, AI will generate prediction algorithms suitable for routine clinical use shortly. The discussion of future medical evidence may be sparked by the findings presented in the studies, which go beyond the investigation of the first targets for data analysis and interpretation, which are potentially expensive, lengthy clinical trials with a constrained patient population that may eventually supplement or even entirely replace real-world data-driven risk assessments.

Solanki et al. [185] presented go-beyond approaches that provide guidelines based on principles such as adherence to “fairness” and adopting a framework based on solutions that AI programmers can use to operationalize ethics in AI for healthcare across all phases of the AI lifecycle, including data management, model development, deployment, and monitoring. The authors strongly emphasize actionable, technical, or quasi-solutions that AI developers can use.

The study presented by Biswas et al. [102] uses an AtheroEdge^TM^ device from AtheroPoint^TM^ to deliver a unique, reliable, and clinically-viable solution to cIMT measurements. The DL approach is used by the system to partition lumen-intima LI-far and MA-far to measure cIMT according to an intelligence-based paradigm. For the distant wall of the carotid artery’s final border extraction, the system applies an ML-based joint coefficient approach to fine-tune this. Data preparation employs a multiresolution paradigm to lighten the computational load. All measurements use an adjusted version of the industry standard polyline distance method. Compared to earlier research, the system performs better.

Aijaz et al. [71] proposed a study that employed a deep-learning classification strategy to categorize the five types of psoriasis and healthy skin. Five different types of psoriasis can develop: plaque, guttate, inverted, pustular, and erythroderma. After the features of color, texture, and form have been extracted, the convolution neural network (CNN) and long short-term memory (LSTM) have been employed. An accuracy rate of 84.2% was shown when CNN and LSTM were employed. Siy et al. [186] introduced a model consisting of a CNN algorithm with different softmax layers to be deployed to obtain higher accuracy. The results obtained show how dependable and efficient the suggested deep learning application is. The consequences of future action investigation into the proposed and current deep learning application could result in the improvement of conventional techniques in biomedical imaging [187]. Moreover, studies on the psoriasis area and severity index in the future will also be possible to score (PASI).

Ali et al. [188] presented study of renal medicine will change as a result of the applications of regenerative medicine, nanotechnology, genomics, artificial intelligence, 3D organ bioprinting, and smartphone applications. Undoubtedly, this will benefit patients’ results and the healthcare system. These improvements are on the way, but they will also bring new difficulties, such as excessive expenses and numerous ethical dilemmas.

Viswanathan et al. [189] explained diabetes exacerbated the development of atherosclerotic plaque. Risk evaluation includes several factors in addition to the degree of vascular stenosis. Plaque vulnerability is influenced by its form, kind, composition, location, TPA, and TPV. The potential for better risk assessment and illness treatment is increased when imaging modalities are added to conventional risk variables. To assist doctors to choose the best interventions for their diabetic patients, screening may thus prove to be crucial. Compared to conventional risk calculators, the 10-year integrated risk calculators and image-based phenotypes produce more accurate risk projections, necessitating more research in the reduction of overall morbidity and death.

A deep learning-based approach is suggested for PD identification that uses voice patterns. The dynamic articulation transition features and the bidirectional LSTM model are creatively combined in the proposed method to record the time-series properties of continuous speech signals. The experimental results demonstrated that the proposed approach significantly outperforms conventional machine learning models using static features in terms of the accuracy of PD detection under the two evaluation methods of 10-fold cross-validation (CV) and splitting the dataset without sample overlap of one individual [190].

Kamble et al. [191] indicated that when four ML models are used in a dataset that has undergone mathematical processing, three different types of digitalized spiral drawing tests have a significant impact on the classification of PD patients versus healthy controls. Results are based on a 40-patient, tiny, unbalanced dataset. The work presented a data set of spiral drawing images with features. Four ML algorithms were used, and an accuracy rate of 98.1% was achieved. Therefore, future PD diagnosis can be carried out with the support of an extended dataset and an extended computational model to help healthcare research on other neurodegenerative disorders. Our review had several studies that explored the AI model’s relationship with the diagnosis and treatment of various diseases. However, no such article was located that addressed all of the components in our analysis.

### 8.3. A View for the Future

A lot of people are concerned that AI may lead to the automation of jobs and a considerable loss of labor, and this concern has garnered a lot of attention. According to research conducted jointly by Deloitte and the Oxford Martin Institute, AI may be responsible for the loss of 35% of jobs in the United Kingdom within the next 10 to 20 years [192]. The loss of employment may be mitigated by several external factors other than technology. These factors include the price of automation technologies, the size and cost of the labor market, the advantages of automation beyond basic labor substitution, and legislative and social acceptance. These factors may keep the actual number of jobs lost to 5% or fewer [193].

To the best of our knowledge, no employment in health care has been eliminated by AI. The limited penetration of AI into the sector thus far, as well as the difficulties of integrating AI into clinical workflows and EHR systems, has contributed to the lack of job impact [194]. It appears that the healthcare positions most likely to be automated are those involving digital information, such as radiography and pathology, rather than those involving direct patient interaction. However, even in positions such as radiologist and pathologist, AI adoption is likely to be delayed. Even though, as we have shown, technologies such as deep learning are making strides into the ability to analyze and classify images, there are many reasons why radiology professions, for example, will not go away anytime soon [195].

For automated image analysis to gain popularity, significant medical regulation and health insurance changes will be required. Pathology and other digitally related elements of medicine have similar causes [196]. As a result, we are unlikely to witness significant changes in healthcare employment as a result of AI during the next 20 years or so. There is also the chance of new employment being established to work with and improve AI technologies. However, static or increasing human employment means that AI technologies are unlikely to significantly cut the costs of medical diagnosis and treatment throughout that timeframe [197].

It also appears increasingly evident that AI systems will not wholly replace human clinicians, but will supplement their efforts to care for patients. Human therapists may eventually shift toward activities and job designs that require distinctly human skills, such as empathy, persuasion, and big-picture integration. Those healthcare professionals that refuse to collaborate with artificial intelligence may be the only ones who lose their employment over time.

### 8.4. Strength, Weakness, and Extensions

Several benefits have been found from doing this review. Our two practical and cost-effective economic models for diagnosis and therapy are our primary strengths. We looked at the benefits and drawbacks of several implementation strategies and the amount of time they would take to figure out which would be the most cost-effective. When both the diagnostic and therapeutic models are evaluated, the latter offers greater savings. Policymakers in the AI industry will find the results valid, and the method can be applied elsewhere if the results are comparable. A cost-benefit analysis, however, is warranted if results vary significantly between demographics. Our research adequately clarified why it is essential to develop AI-based devices to gain regulatory market approval and the steps required to obtain FDA 510 (K) clearance for AI-based products.

Our work has several constraints, the most important of which is its limited generality. Variations in the number of people who undergo screening and the cost of employing human graders and specialists will likely provide varying results across countries. On the other hand, our decision tree may be readily modified to represent these alternative possibilities. Second, we may have underestimated the cost savings from the fully automated model due to fewer false referral instances, due to a lack of relevant literature on the corresponding prevalence; we only examined diagnosis and treatment and overlooked other factors. Lastly, this study can be extended for meta-analysis [171].

We anticipate that AI will be an integral part of emerging medical technologies. It is the central capability propelling the growth of precision medicine, which is widely recognized as a welcome improvement in treatment. We expect AI will eventually master the domain of providing diagnosis and treatment suggestions, notwithstanding the difficulty of early attempts. It is conceivable that most radiology and pathology images will be examined by a machine in the future, thanks to the rapid development of artificial intelligence for imaging processing. The use of speech and text recognition for common healthcare tasks, including patient communication and note-taking, is expected to increase.

**Table 2:** Benchmarking of studies.

**C1**	**C2**	**C3**			**C4**		**C5**		**C6**			**C7**	**C8**	**C9**	**C10**	**C11**
**SN**	**Author**	**Country**			**Journal**		**Study Type**		**FoV**			**Objective**	**PS**	**Cli-Val**	**Diagnosis (Invasive/Noninvasive)**	**Treatment (Invasive/Noninvasive)**
1	Smetherman et al. [182] (2021)	USA			Breast Imaging		P.R.		Cancer			Improving the quality of care and/or reducing healthcare costs by using AI	1012	No	Noninvasive	NR
2	Challen et al. [183] (2019)	UK			Artificial intelligence, bias and clinical safety		R.		Clinical safety			To set short and medium ML clinical safety goals	NR	No	Noninvasive	NR
3	Almazán et al. [82] (2019)	Italy			Clinical Pharmacy		P.R.		Renal			Evaluate the effectiveness, safety, and economic cost of nivolumab in real-world clinical practice	221	No	Noninvasive	NR
4	Yuan et al. [184] (2020)	China			Medical Sciences		P.R.		Renal			Challenges in kidney diagnosis and treatment	NR	No	Noninvasive	NR
5	Solanki et al. [185] (2022)	Australia			Operational ethics in AI framework		R		NA			NR	NR	No	Noninvasive	NR
6	Biswas et al. [102] (2018)	India			DL-based strategy for accurate Carotid Intima-Media Measurement		R		Heart			The carotid intima-media thickness (cIMT) is an important biomarker for monitoring cardiovascular disease and stroke	204	No	Noninvasive	NR
7	Siy et al. [186] (2018)	Taiwan			IEEE Conference		R		Skin			DL-based psoriasis detection	5700	No	Noninvasive	NR
8	Aijaz et al. [71] (2022)	Pakistan			Journal of Healthcare Engineering		R		Skin			Effective classification of different psoriasis types using deep learning applications	473	No	Noninvasive	NR
9	Ali et al. [188] (2022)	Iraq			Kidney Diseases Transplantation		P.		Renal			Renal medicine	NR	No	NR	NR
10	Viswanathan et al. [189] (2020)	India			Preventive health check in patients with diabetes		R.		Diabetes			Cost-effective carotid ultrasound screening for diabetes patients	NR	NR	Noninvasive	NR
11	Sarki et al. [198] (2020)	USA			Health Information Science and Systems		P.R.		Diabetes Retinopathy			Deep learning-based automated identification of multiple classes of diabetic eye disorders	1748	NR	Noninvasive	NR
12	Quan et al. [199] (2021)	Japan			IEEE Access		P.R.		Parkinson’s			Using dynamic speech features, a deep learning-based approach for Parkinson’s disease detection	45	NR	Noninvasive	NR
13	Kamble et al. [191] (2021)	India			Measurement and Sensor		P.R.		Parkinson’s			Parkinson’s disease classification using digital spiral drawings	25	NR	Noninvasive	NR
		**C12**							**C13**						**C14**	
**SN**	**Author**	**AI Type**							**Cost Analysis Parameter**						**Outcome of study**	
		**AI Type**	**ACC**	**SEN**	**SPE**	**AUC**	**MCC**	**F1**	**Cost Analysis Parameter**	**Input Modality**	**Model Analysis**	**Screening cost**	**Maintain Cost**	**Cost Savings (USD) Per. Sample**		
1	Smetherman et al. [182] (2021)	NR	NR	NR	NR	NR	NR	NR	NR	Image	Yes	Yes	NR	318	AI could assess individual situations, make appropriate decisions, and aid in the management of renal disease.	
2	Challen et al. [183] (2019)	NR	NR	NR	NR	NR	NR	NR	NR	NR	NR	NR	NR	NR	ML DSS deployment will most likely concentrate on diagnostic decision support. ML Diagnostic decision assistance should be assessed with the same rigors as a novel laboratory screening test.	
3	Almazán et al. [82] (2019)	NR	NR	NR	NR	NR	NR	NR	NR	Point Data	Yes	Yes	NR	61	AI for improved clinical benefit from nivolumab therapy.	
4	Yuan et al. [184] (2020)	NR	NR	NR	NR	NR	NR	NR	NR	Point Data	Yes	Yes	NR	62	Artificial intelligence can consider individual situations, make appropriate decisions, and make significant advancements in the management of renal disease.	
5	Solanki et al. [185] (2022)	NR	NR	NR	NR	NR	NR	NR	NR	NR	Yes	Yes	Yes	Yes	Guidelines, frameworks, and advancement of technologies for ethical AI that reflect human values, such as self-direction, in healthcare.	
6	Biswas et al. [102] (2018)	DL	86.78	0.76	NR	0.86	NR	NR	NR	Image	NR	NR	NR	NR	High-level features are extracted from the CCA US photos using CNN’s 13 layers. To produce clear and crisp segmented images, these features were upsampled using FCN upsampling layers, and the skipping operation was carried out.	
7	Siy et al. [186] (2018)	DL	91.5	NR	NR	NR	NR	NR	NR	Image	NR	NR	NR	NR	A DNN-based psoriasis detection presented having 91.5% accuracy.	
8	Aijaz et al. [71] (2022)	DL	84.2	0.81	0.71	NR	NR	NR	NR	Image	NR	NR	NR	NR	This study employed a CNN-based deep learning classification strategy to categorize the five types of psoriasis.	
9	Ali et al. [188] (2022)	NR	NR	NR	NR	NR	NR	NR	NR	NR	NR	NR	NR	NR	Machine learning and artificial intelligence have ushered in a new era in medicine and nephrology.	
10	Viswanathan et al. [189] (2020)	NR	NR	NR	NR	NR	NR	NR	NR	Image	NR	NR	NR	14	Diabetes exacerbated the deposition of atherosclerotic plaque. Risk assessment includes other factors in addition to the degree of vessel stenosis.	
11	Sarki et al. [198] (2020)	DL	84.88	0.87	NR	NR	NR	NR	NR	Image	NR	NR	NR	NR	The development of moderate and multi-class DL algorithms for the automatic detection of DED, according to the British Diabetic Association (BDA) criteria.	
12	Quan et al. [199] (2021)	DL	80.90	0.87	0.92	0.83	0.53	NR	NR	Speech	NR	NR	NR	NR	The dynamic articulation transition features and the bidirectional LSTM model are combined ingeniously in the proposed method to record the time-series properties of continuous speech data.	
13	Kamble et al. [191] (2021)	ML	91.6	NR	NR	NR	NR	0.8	NR	Image	NR	NR	NR	NR	Digitalized spiral drawing tests significantly affect how PD patients and healthy controls are classified.	

## 9. Conclusions

Current research examines the impact of AI in health care moderately, and reveals qualitative flaws in methodology. This study provides a clear explanation of the diagnostic and therapeutic paradigm needed for future cost-effectiveness analyses. The presented study delineated the motivation for building AI-based products for successful regulatory market approval and the necessary element for AI-based products to undergo successful FDA 510 (K) approval. They should contain the original expenditure, ongoing costs, and a comparison to alternative technology. This way, a complete and segmented cost-benefit analysis may be offered, which will serve as a solid basis for making decisions about AI installations.

From a strategic point of view, cost-effectiveness studies were assessed using a quality criteria catalog. Because decisions are not solely based on medical improvement rates, the business management decision making basis has been identified as critical for favorable implementation decisions and subsequent wide-scale applications. The integration of the business management perspective encompasses not only the conventional cost considerations, such as one-time and continuing costs, but also the options for delivering cutting-edge healthcare solutions in various ways.

## Figures and Tables

**Table 1:** Types of Food and drug administration (FDA) approvals for AI/ML-based healthcare technology are described [177].

SN	FDA Approval Stages	Description
1	510 (k) clearance	A 510 (k) authorization is granted to an algorithm if it is at least as secure and effective as another equivalent, commercially available algorithm. Alongside the claim, the applicant for this clearance must provide substantial proof of equivalence. It is illegal to commercialize the algorithm that is awaiting approval until it has been determined to be reasonably comparable to the other algorithm.
2	Premarket approval	For Class III medical devices, algorithms receive premarket approval. The safety and efficacy of the latter are assessed through more comprehensive scientific and regulatory processes since they can have a significant impact on human health. The FDA must find sufficient scientific evidence supporting the device’s usefulness and safety before approving an application. The applicant can move further with product marketing after receiving approval.
3	de novo pathway	The de novo category is used to categorize novel medical devices with sufficient safety and efficacy and with broad controls, but in which there are no lawfully marketed equivalents. Before approving and permitting the devices to be marketed, the FDA conducts a risk-based evaluation of the device.

## Data Availability

No data availability.

## Appendix A

**Table A1:** Cost Effective analysis for the diagnosis.

Categories	Count	Years									
		1	2	3	4	5	6	7	8	9	10
Patient Size per hospital per year	3650	7300	9125	10,950	12,775	14,600	16,425	18,250	20,075	21,900	23,725
No. of Hospital	20	20	22	24	26	28	30	32	34	36	38
Per day Patient Per hospital	20	20	25	30	35	40	45	50	55	60	65
Total patient	73,000	2,920,000	5,018,750	7,884,000	1,162,5250	1,635,2000	22,173,750	29,200,000	37,540,250	47,304,000	58,600,750
**Conventional Method**											
Physician charges per hour	500	500	550	605	665.5	732.05	805.255	885.7805	974.3586	1071.794	1178.974
Conventional method time (minutes) per day	60	1200	1500	1800	2100	2400	2700	3000	3300	3600	3900
Conventional method time (hours) per day	1	20	25	30	35	40	45	50	55	60	65
Physician charges per day in USD		10,000	13,750	18,150	23,292.5	29,282	36,236.48	44,289.03	53,589.72	64,307.66	76,633.3
Physician Charges per year per hospital		3,650,000	5,018,750	6,624,750	8,501,763	10,687,930	13,226,313	16,165,494	19,560,248	23,472,297	27,971,154
**AI-based Method**											
Physician charges per hour in USD	500	500	550	605	665.5	732.05	805.255	885.7805	974.3586	1071.794	1178.974
AI-based system time (minutes) per day	60	1000	1225	1440	1645	1840	2070	2300	2530	2760	2990
AI-based system time in (hours) per day	1	16.66667	20.41667	24	27.41667	30.66667	34.5	38.33333	42.16667	46	49.83333
Physician charges per day in USD		8333.333	11,229.17	14,520	18,245.79	22,449.53	27,781.3	33,954.92	41,085.45	49,302.54	58,752.2
Physician charges per year per hospital in USD		3,041,667	4,098,646	52,99,800	6,659,714	8,194,080	10,140,174	12,393,545	14,996,190	17,995,428	21,444,552
**Difference (Conventional–AI)**											
Saving in time (minutes) per day		200	275	360	455	560	630	700	770	840	910
Saving in time (hours) per day		3.333333	4.583333	6	7.583333	9.333333	10.5	11.66667	12.83333	14	15.16667
Saving in Physician charges per day in USD		1666.667	2520.833	3630	5046.708	6832.467	8455.178	10,334.11	12,504.27	15,005.12	17,881.1
Saving in Physician charges per year per hospital in USD		608,333.3	920,104.2	1,324,950	1,842,049	2,493,850	3,086,140	3,771,949	4,564,058	5,476,869	6,526,603

**Table A2:** Cost Effective analysis for the treatment.

Categories	Count	Year									
		1	2	3	4	5	6	7	8	9	10
Patient Size per hospital per year	3650	3650	5475	7300	9125	10,950	12,775	14,600	16,425	18,250	20,075
No. of Hospital	20	15	17	16	18	17	19	18	20	19	21
Per day Patient Per hospital	20	10	15	20	25	30	35	40	45	50	55
Total patient	73,000	547,500	1,396,125	2,336,000	4,106,250	5,584,500	8,495,375	10,512,000	14,782,500	17,337,500	23,186,625
**Conventional Method**											
Physician charges per hour	1000	1000	1100	1210	1331	1464.1	1610.51	1771.561	1948.7171	2143.58881	2357.947691
Conventional method time (minutes) per day	180	1800	2700	3600	4500	5400	6300	7200	8100	9000	9900
Conventional method time (hours) per day	3	30	45	60	75	90	105	120	135	150	165
Physician charges per day in USD		30,000	49,500	72,600	99,825	131,769	169,103.55	212,587.32	263,076.80	321,538.32	389,061.36
Physician Charges per year per hospital		10,950,000	18,067,500	26,499,000	36,436,125	48,095,685	61,722,795.75	77,594,371.8	96,023,035.1	117,361,487.3	142,007,399.7
**AI-based Method**											
Physician charges per hour in USD	1000	1000	1100	1210	1331	1464.1	1610.51	1771.56	1948.71	2143.58	2357.94
AI-based system time (minutes) per day	90	500	735	960	1175	1380	1610	1840	2070	2300	2530
AI-based system time in (hours) per day	1.3	8.33	12.25	16	19.58	23	26.83	30.66666667	34.5	38.33	42.16
Physician charges per day in USD		8333.33	13,475	19,360	26,065.41	33,674.3	43,215.35	54,327.87067	67,230.73	82,170.90438	99,426.79
Physician charges per year per hospital in USD		3,041,666.66	4,918,375	7,066,400	9,513,877.08	1,229,1119.5	1,577,3603.36	19,829,672.79	24,539,220.08	29,992,380.1	36,290,779.92
**Difference (Conventional–AI)**											
Saving in time (minutes) per day		1300	1965	2640	3325	4020	4690	5360	6030	6700	7370
Saving in time (hours) per day		21.66	32.75	44	55.41	67	78.16	89.33	100.5	111.66	122.83
Saving in Physician charges per day in USD		21,666.66	36,025	53,240	73,759.58	98,094.7	125,888.19	158,259.44	195,846.068	239,367.41	289,634.57
Saving in Physician charges per year per hospital in USD		7,908,333.33	131,49,125	19,432,600	26,922,247.92	35,804,565.5	45,949,192.39	57,764,699.01	71,483,815.02	87,369,107.25	105,716,619.8

**Table A3:** AI content considered for cost analysis.

*SN*	Category	Content
**X1**	Data collection	Patient size per hospital
		Enrollment cost per patient
**X2**	Engineering R&D cost	Data verification
		Data validation
		Scientific algorithms
		Graphical user interface (design)
		Cloud/storage
		Software technology updation
		Hardware technology updation
		Prototype testing
		Maintenance and support
**X3**	Human resource cost	ML scientist
		DL scientist
		Verification and validation scientist
		Clinical scientist
		Database engineer
		Graphical user interface engineer
		System administrator
		Cloud engineer
		Marketing professional
		Secretary
**X4**	Commercialization cost	FDA 5K approval
		Regulatory costs of various countries
		Release cost
**X5**	Marketing cost	Marketing
		Technical marketing
		Installation
**X6**	Infrastructure cost	Office space
		Furniture
		Hardware
		Software
		Electricity
